# Drug-Induced Liver Injury

**DOI:** 10.1155/2017/2461694

**Published:** 2017-01-09

**Authors:** Eileen E. N. Almario, Jürgen Borlak, Ayako Suzuki, Minjun Chen

**Affiliations:** ^1^Office of Computational Science, Center for Drug Evaluation and Research, U.S. Food and Drug Administration, Silver Spring, MD, USA; ^2^Center of Pharmacology and Toxicology, Hannover Medical School, Hannover, Germany; ^3^Department of Medicine, University of Arkansas for Medical Sciences, Little Rock, AR, USA; ^4^Division of Bioinformatics and Biostatistics, National Center for Toxicological Research, U.S. Food and Drug Administration, Jefferson, AR 72079, USA

 Among acute hepatic injuries drug-induced liver injury (DILI) is rare; nonetheless it is one of the most common causes for withdrawal of drugs from the market. Progress has been made in the development of tools to identify the risk for DILI once it occurs in clinical trials. Recent efforts defined drug properties, for example, reactive metabolites, lipophilicity, and therapeutic dose which were found to correlate with the hepatotoxicity potential of chemical entities. More work is needed, however, to understand how to predict liver injury for individual patient.

This special issue addresses current needs for an improved understanding of DILI and highlights the importance for a systematic, collaborative linking of in vitro, preclinical, and clinical evidence data to enhance DILI prediction. A total of 17 submissions contributed to this special issue with 7 manuscripts published, including three reviews discussing the challenges of in vitro models, engineered liver models, and herbal hepatotoxicity, followed by two studies in zebrafish models and two mechanistic studies of hepatotoxicity induced by Cyclosporine A and valproic acid.

In a paper coauthored with colleagues from the regulated industry, F. A. Atienzar et al. review the critical challenges and opportunities associated with the use of in vitro models for predicting human DILI. Specifically, they emphasize lack of standardization as the major challenge and describe some practice issues (e.g., DILI classification, cut-off concentration for in vitro assay, and endpoint selection) that can further improve the development of such models. They highlight the need for an integrated approach in assessing toxicity, particularly for idiosyncratic DILI which is less reliably predicted by current tools, citing successful use of in vitro models for toxicity prediction in other domains (e.g., proarrhythmia risk).

C. Lin and S. R. Khetani systematically review recent technological progress in developing liver models, including micropatterned cocultures, spheroidal and bioprinted cultures, perfused biochips, precision cut liver slices, and humanized rodents together with high-content assays and in silico prediction. They highlight the challenges in developing a realistic model and the selection of measures and endpoints that correspond to phenotypic DILI in humans. They conclude that advances in engineered liver models will enable better prediction of toxicity and an understanding of idiosyncratic DILI to eventually reduce drug attrition, animal usage, and DILI risk in humans.

With the growing use of Chinese Herbal Medicine (CHM) worldwide, the implication of associated DILI needs to be considered. C. Liu et al. systematically summarize the historical literature and current scientific knowledge on herbal toxicity and reiterate safety regulation of herbal medicine in China. They emphasize the importance of accurate diagnosis and treatment of herb-induced liver injury and specifically discuss the contents and important implications of the recently released Chinese guideline for diagnosis and treatment for herb-induced liver injury.

Valproic acid (VPA) has been marketed for nearly 40 years although its mechanism of action is still not fully understood. Warnings and contraindications have been endorsed to reduce adverse drug reactions which may include serious or life threatening liver injury. R. Chang et al. examined the effect of VPA on oleic acid-induced hepatocyte steatosis in the FL83B cell line and found VPA to enhance oleic acid-induced lipid droplet accumulations in a dose-dependent manner. They found that VPA triggered PPAR*γ* nuclear translocation to endorse lipogenesis rather than lipolysis and to increase expression of the cell surface fatty acid transporter CD36, thus further augmenting VPA-induced hepatic steatosis.

A. Korolczuk et al. report a study with Cyclosporine A, that is, an immunosuppressive drug that revolutionized transplantation medicine some 30 years ago. After 28 days of drug treatment, they found that impaired liver function was associated with ultrastructural damage in mitochondria accompanied by significant changes in oxidative stress markers and lipid peroxidation products. Based on these observations, they suggested that oxidative stress and mitochondria damage might play a crucial role in the course of Cyclosporine A induced hepatotoxicity.

Zebrafish is a promising model in the assessment of DILI but its utility needs further exploration. D. Cheng et al. employ a biomolecular imaging approach that provides a full image set for ultrastructural mapping of the zebrafish larvae gastrointestinal system. They conclude that this imaging approach could be used for studying various digestive disorders and drug delivery pathways in the zebrafish.

In another study by H.-S. Nam et al., zebrafish exposed to escalating doses of tamoxifen were found to express miRNA-122 in the liver but not in other organs. Histological changes and tamoxifen blood concentration varied in a dose-dependent pattern similar to acetaminophen exposure. The authors conclude that miRNA-122 might be a potential marker for acute liver toxicity in zebrafish.

These selected articles portend well for the translation of basic science findings into tools that enhance DILI prediction, enable directed therapy to minimize harm, and preserve the availability of therapies in patients that are likely to benefit.

